# Validation of a New Ankle Brachial Index Measurement System Using Pulse Wave Velocity

**DOI:** 10.3390/bios14050251

**Published:** 2024-05-16

**Authors:** Juan David Romero-Ante, Esther Chicharro-Luna, Juliana Manrique-Córdoba, José María Vicente-Samper, Alba Gracia-Sánchez, José María Sabater-Navarro

**Affiliations:** 1 Neuroengineering Biomedical Group, Medical Robotics Unit, Institute of Bioengineering, Miguel Hernández University of Elche, 03202 Elche, Spain; 2 Department of Behavioural Sciences and Health, Nursing Area, Faculty of Medicine, Miguel Hernández University of Elche, 03550 San Juan de Alicante, Spain

**Keywords:** peripheral artery disease, ankle brachial index, pulse wave velocity, electrocardiogram, photoplethysmography, blood pressure wave

## Abstract

Peripheral artery disease (PAD) is a common circulatory disorder characterized by the accumulation of fats, cholesterol, and other substances in the arteries that restrict blood flow to the extremities, especially the legs. The ankle brachial index (ABI) is a highly reliable and valid non-invasive test for diagnosing PAD. However, the traditional method has limitations. These include the time required, the need for Doppler equipment, the training of clinical staff, and patient discomfort. PWV refers to the speed at which an arterial pressure wave propagates along the arteries, and this speed is conditioned by arterial elasticity and stiffness. To address these limitations, we have developed a system that uses electrocardiogram (ECG) and photoplethysmography (PPG) signals to calculate pulse wave velocity (PWV). We propose determining the ABI based on this calculation. Validation was performed on 22 diabetic patients, and the results demonstrate the accuracy of the system, maintaining a margin of ±0.1 compared with the traditional method. This confirms the correlation between PWV and ABI and positions this technique as a promising alternative to overcome some of the limitations of the conventional method.

## 1. Introduction

Peripheral artery disease (PAD) is a common circulatory disorder caused by atherosclerosis, a condition characterized by the buildup of fats, cholesterol, and other substances in the arteries and on their walls. This buildup causes the peripheral arteries to narrow, reducing blood flow from the heart to other parts of the body. PAD primarily affects the arteries of the lower extremities, causing reduced blood flow to the legs and feet. It can also affect the arteries that supply blood to the head, arms, and kidneys [1].

PAD affects approximately 6% of the adult population worldwide, and its prevalence continues to increase. Typically recognized as asymptomatic, PAD commonly manifests as muscle pain with walking that tends to resolve with rest. However, in advanced stages, symptoms may include rest pain, ulceration, and gangrene, with the potential risk of amputation if not managed appropriately [2]. Individuals with this condition have a significantly increased risk of developing cardiovascular disease and cerebrovascular events [3]. Smoking, diabetes mellitus (DM), arterial hypertension, and hypercholesterolemia have been identified as critical factors in the development of PAD [4]. Impaired and inadequate glycemic control is associated with PAD, although these patients may be asymptomatic due to the coexistence of neuropathy [5].

In the clinical setting, PAD is identified by healthcare professionals by observing signs during physical examination. Key indicators include a weakened pulse or evidence of impaired wound healing. For accurate diagnosis of PAD, ankle brachial index (ABI) testing has been shown to be a highly reliable and valid assessment, especially when performed using Doppler ultrasound [6]. The ABI is a widely used non-invasive clinical test. The procedure involves placing blood pressure cuffs on the brachial arteries and around each ankle, specifically over the malleoli (between the tibia and fibula). The patient is initially placed in the supine position for a resting period of 5 to 10 mins. Systolic blood pressure (SBP) is then recorded in each brachial artery and lower extremity using a manual Doppler probe [7]. ABI is performed on both sides of the body, so SBP measurements are taken on the right and left arms and ankles, respectively. The result is obtained as the ratio between the SBP measured at the ankle and at the arm [8]. Arterial pressures in the ankle region are usually higher than brachial pressures measured in the arms; hence, ABI ≤ 0.9 is used as a diagnostic criterion for PAD [9]. An ABI between 0.9 and 1.3 is considered normal [8,9], while values above 1.4 may be associated with an increased risk of stroke and heart failure [10].

The ABI measurement procedure has some drawbacks, such as the time required to perform it, the need for Doppler equipment, and the need for prior training of the clinical staff in charge to minimize the variability of the results. This has led to the exploration of alternative diagnostic methods for clinical practice. For example, the absence or weakness of pulses in the distal regions of the lower extremities was initially thought to be associated with PAD; however, some studies have refuted this hypothesis due to the lack of positive results [11]. Another method of measuring ABI has been to use an automated sphygmomanometer to record SBP. However, comparative studies between this measurement and that obtained by the traditional method did not show significant agreement, so this technique was discarded. In addition, the use of automated sphygmomanometers involves the occlusion of blood flow through the cuff, which could cause anxiety and discomfort in the patient, leading to unreliable measurements, especially in individuals with hypertension [12].

Thus, photoplethysmography (PPG) is emerging as a simple and non-invasive optical technique capable of detecting variations in blood volume caused by the expansion and contraction of arteries in response to the arterial pressure wave. Exploratory studies have demonstrated a correlation between blood pressure measurements obtained with Doppler devices and those obtained with PPG. In [13], the results of one of the first studies conducted in 1998 are presented, in which a correlation coefficient of 0.875 was obtained in ABI measurements performed on 52 limbs. This suggested that the PPG technique could be a viable replacement for Doppler equipment in the development of automated ABI measurement systems. The results of other studies have supported this hypothesis, with variation intervals ranging [−0.27, 0.26]; considering the discrepancy between measurements obtained with the traditional method and PPG-based systems, it is concluded that the PPG technique is easily implementable in general screening tests for PAD [14,15,16].

On the other hand, pulse wave velocity (PWV) is characterized as the rate at which the arterial pressure pulse travels through the circulatory system. This velocity can be estimated using the parameter pulse transit time (PTT), which is the time it takes for a pulse wave to travel between two points in the cardiovascular system. PTT can be derived from both electrocardiogram (ECG) and PPG signals [17]. This parameter can be used to define the ankle brachial pulse wave velocity (baPWV), an important measure for detecting cardiovascular risk and assessing the severity of atherosclerotic vascular damage [18]. Research has shown that baPWV correlates with the assessment of elasticity of both large and small arteries, highlighting its enhanced predictive value for hypertension [19]. The measurement of baPWV and ABI has been associated with the diagnosis of several diseases [20,21]. It would be interesting to explore the possibility of establishing a measure of ABI from PWV, as the latter is used as a method of assessing arterial stiffness, which is associated with the risk of developing atherosclerosis.

This article presents a system capable of simultaneously measuring ECG and PPG signals. The system is capable of determining heart rate variability (HRV) and PTT using characteristic points from the ECG and PPG signals. With this information, a method is developed to calculate PWV, considering the approximate length of the arteries that carry blood from the heart to the index finger of the hand and the hallux toe, proposing to determine the ABI in this way. This system represents an interesting alternative that could mitigate the complications associated with the conventional ABI measurement procedure in the evaluation of patients with PAD. In the Materials and Methods section, the electronic devices designed for the acquisition of ECG and PPG signals, as well as the data processing and proposed methodology for the calculation of the ABI, are described in detail. The results section presents the results of the system validation and discusses the accuracy compared with the standard procedure using Doppler devices.

## 2. Materials and Methods

### 2.1. Physiological Signals and Biomedical Parameters

The system developed in this study uses simultaneously measured ECG and PPG signals with biomedical parameters such as PPT and PWV to determine the ABI. These signals and parameters are described below.

#### 2.1.1. Electrocardiogram (ECG)

An electrocardiogram (ECG) is a direct recording of the heart’s electrical activity, which reflects the heart’s excitement as it contracts and relaxes with each heartbeat. This painless test is performed by placing two or more electrodes on the patient’s skin at different points around the chest. The ECG plays a key role in diagnosing heart disease and assessing heart health [22]. For example, the R-R interval, which is the distance between two consecutive R waves, defines an individual’s heart rate [23].

#### 2.1.2. Photoplethysmography (PPG)

Photoplethysmography (PPG) is a simple and non-invasive optical technique used to monitor peripheral heart rate by detecting changes in blood volume through the skin. There are two main configurations for characterizing the blood pressure waveform using PPG. In the first, the LED diode is placed on one side of the tissue while the photodetector is placed on the other side to measure light; in this case, the voltage signal is inversely proportional to blood flow. In the second configuration, both the LED diode and the photodetector are placed in the same measurement area. During systole, as the pressure increases, the blood concentration increases, and so does the light absorption. The opposite occurs during diastole, where blood pressure decreases and light absorption decreases [24]. These changes in blood volume during the cardiac cycle allow blood pressure to be represented as a periodic signal in which systolic and diastolic peaks are identified. The systolic peaks are used to determine heart rate considering the time interval between them [25].

#### 2.1.3. Pulse Wave Velocity (PWV)

Pulse wave velocity is defined as the speed at which the blood pressure pulse travels through the circulatory system. PWV is calculated as the distance traveled by the pulse wave divided by the time it takes to travel that distance. It is used as a measure of arterial stiffness and allows the diagnosis of cardiovascular disease [26]. To calculate PWV, it is necessary to know the time it takes for the pressure wave to travel a distance and the length of the distance. The former can be approximated by the parameter pulse transit time (PTT), which determines the time it takes for the pulse to travel between two points in the circulatory system. In particular, the behavior of PTT serves as a parameter for monitoring changes in blood pressure [27]. PTT can be obtained from the R-peaks of the ECG signal and the systolic amplitude maxima of the PPG waveform, which is usually measured through the fingertips. PTT can also be estimated from the Q-points of the ECG and the onset of the systolic rise in the PPG [17,28].

On the other hand, the length of the arteries is not an easily known parameter; it varies from individual to individual and complicates the PWV measurement procedure. Studies have been developed to automatically adjust for the length of arteries supplying blood to the extremities. In [18], equations were obtained to estimate the length of the brachial artery and the artery that carries blood flow to the ankle, which could be related to the tibial artery.

### 2.2. Measuring Devices

The system consists of two devices. The first captures the ECG signal in the patient’s chest and the PPG signal in the index finger of the hands. The second device records the PPG signal at the hallux. The devices were designed using a MAX86150 module from Analog Devices [29] and a Teensy 4.0 development board from PJRC [30] to acquire the signals. The design of the devices is described below.

#### 2.2.1. MAX86150

Maxim Integrated’s MAX86150 biosensor module enables ECG and PPG measurements from a single device with a 16-bit resolution. This device consists of LEDs, photodetectors, and an analog front end to provide high performance and accuracy in obtaining PPG and ECG measurements. It is also FDA (United States Food and Drug Administration)-cleared, which supports its reliability and accuracy in various applications. Its compact design and low power consumption make it easy to integrate into devices such as smartphones and laptops.

In terms of features, this device stands out for its ability to obtain ECG signal derivation with only two electrodes. PPG signals are captured by an encapsulation that integrates LEDs and photodetectors, which simplifies the acquisition of measurements in the same region. It also features an instrumentation amplifier with a high common mode rejection ratio (CMRR). With a miniature design (3.3 mm × 5.6 mm × 1.3 mm), this optical module operates from a typical 1.8-volt (V) supply, while the LEDs responsible for the infrared light require a separate 3.3 V supply. Communication with the integrated circuit is conducted via the inter-integrated circuit (I2C) protocol. It should be noted that at the input of each ECG electrode, according to the manufacturer’s specifications, a low-pass R-C filter with a cutoff frequency of approximately 318 Hz is implemented, which ensures that the frequency spectrum of the ECG signal is obtained [31].

#### 2.2.2. Teensy 4.0 Board

The devices are equipped with a Teensy 4.0 development board, which plays a central role in the signal acquisition phase. This module is characterized by high performance and versatility, making it ideal for applications in robotics, instrumentation and control, user interfaces, Internet of Things (IoT), and others.

The Teensy 4.0 features a microcontroller that integrates a 600 MHz ARM Cortex-M7 processor, 1984 kilobytes of flash memory, and 1024 kilobytes of RAM. It requires a typical supply voltage of 5.0 V and operates at 3.3 V. Among its communication interfaces, it has three I2C ports necessary for communication with the MAX86150 module, as well as a USB port that facilitates the transfer of acquired data to a central computer responsible for its management. An important advantage of these microcontrollers is their compatibility with the Arduino programming environment, thanks to the Teensyduino extension. This simplifies the implementation of libraries and codes developed for Arduino. In this case, the ProtoCentral MAX86150 PPG and ECG IC library [32] is used.

#### 2.2.3. Signal Acquisition

The system consists of one device to measure the ECG signal on the chest and PPG on the index fingertip of the hand and another device to measure the PPG signal on the hallux fingertip. A printed circuit board (PCB) was fabricated for each device. These boards integrate the Teensy 4.0 development board and the MAX86150 biosensor. The circuit designs were based on the sensor data sheet specifications and included a 1.8 V voltage regulation circuit to power the optical module. In addition, the signal sampling frequency was set to 200 Hz.

In each device, the MAX86150 module acts as a slave to the Teensy 4.0 microcontroller to simplify the transfer of acquired data. In addition, the control module sends the information via the universal asynchronous receiver–transmitter (UART) serial communication protocol to a central computer responsible for storing and managing the data for further analysis. The Teensy is connected to the computer via the USB interface, which facilitates the transfer of information and provides the 5 V power supply.

**Figure 1:**
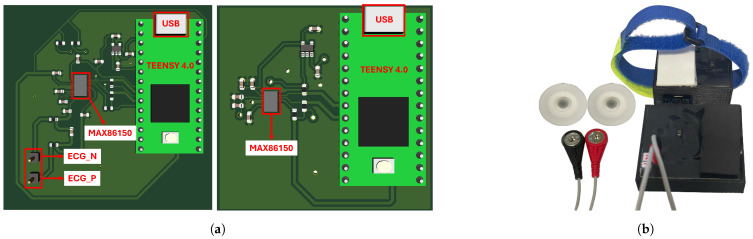
(**a**) The three-dimensional layout of the designed boards; (**b**) measuring devices.

Figure 1a shows the three-dimensional layout of the circuit boards. On the left is the PCB for the first unit, with the position of the optical module for capturing the PPG signal highlighted, along with the connectors for the cables used in the ECG measurement, identified as ECG_P and ECG_N. The PCB for the second unit is shown on the right. Figure 1b shows the manufactured measurement devices, highlighted for their compact size, equipped with housings to improve fixation and comfort. The picture also shows the connection of the cables and electrodes used for the ECG signal. To reduce the signal noise emission, the circuit boards were covered with insulating tape. Velcro was also used to attach the PPG meter to the hallux.

**Figure 2:**
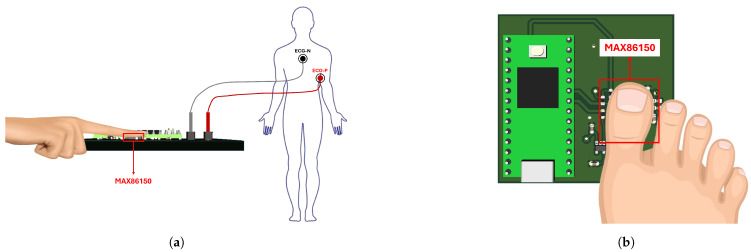
(**a**) Index finger position and electrodes connection; (**b**) hallux toe position.

In addition, Figure 2 shows the placement of the index finger and hallux over each optical module to capture the PPG signals. It is important to completely cover the sensor area to minimize interference from external light. For the ECG signal, the position of each electrode is observed, with the N terminal placed on the individual’s chest and the P terminal being in the axillary region, as shown in the diagram.

### 2.3. Signal Processing

**Figure 3:**
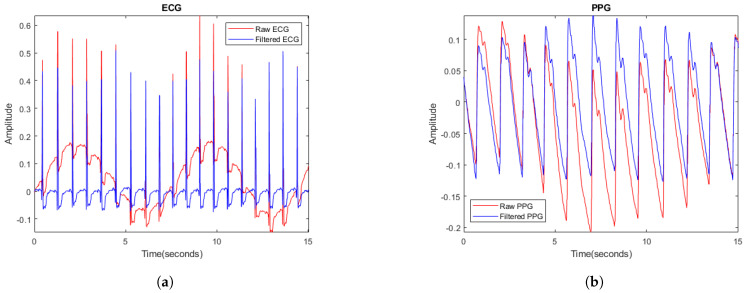
(**a**) Raw ECG signal in red and filtered signal in blue; (**b**) raw PPG signal in red and filtered signal in blue.

The information captured by the measuring devices is collected and stored in text files on a computer. The data are processed and analyzed using Matlab software [33]. Although the MAX86150 optical modules incorporate protection and rejection circuitry to ensure accurate measurements, it is essential to include an initial filtering stage to eliminate potential electromagnetic interference. These interferences can manifest themselves, for example, as an alternating component due to noise emitted by the power grid. A high-pass filter with a cutoff frequency of 50 Hz is set up for both signals. Figure 3 illustrates the effect of filtering: the unfiltered signals are shown in red, while the filtered signals are shown in blue. The elimination of the alternating wave associated with the electric current is evident, resulting in a significant improvement in the clarity of the filtered signals.

In order to improve the quality of the signals, a second processing stage was implemented to eliminate noise generated by the user’s movements or potential cardiac problems that may manifest themselves in the ECG signals. For this purpose, a time–frequency analysis was performed using the Matlab Wavelet Toolbox [34].

**Figure 4:**
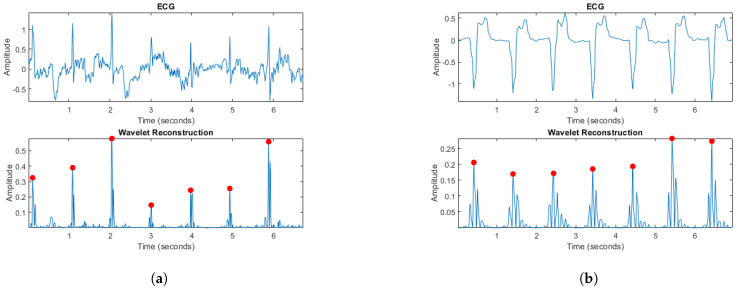
(**a**) ECG signal with variation and noise and result of wavelet processing; (**b**) ECG signal with negative QRS complex and result of wavelet processing. The points highlighted in red indicate the identified R-peaks.

For the ECG signal, a four-level decomposition was performed using the fourth-order wavelet of the Symlets family ‘sym4’, which is characterized by its similarity to the QRS complex, making it an ideal choice for R-peak detection [35]. In the wavelet transform, the information contained in the high frequencies is analyzed with greater precision at a smaller scale, while it is focused on the low frequencies at a larger scale. In this context, the results of the detail coefficients 2 and 4 of the decomposition are used. Coefficient 2 contains high-frequency information that allows us to identify the QRS complex and emphasize the R-peak of the signal. On the other hand, coefficient 4 contains low-frequency information, which in some cases facilitates the representation of P- and T-waves in the ECG signal. This reconstruction procedure makes it possible to characterize the signals obtained in people with a weak pulse, where it may be difficult to recognize the R-peak, or in cases where the ECG derivation obtained represents the QRS complex negatively. Figure 4a shows the original signal, characterized by significant variation and noise that make it difficult to identify the components of the ECG signal, together with the result of the reconstruction using the wavelet transform. Figure 4b shows another signal with the QRS complex in the negative, together with the result of the processing performed. The points highlighted in red indicate the identified R-peaks.

In the case of the PPG signals, the wavelet transform is used to smooth the signal obtained in the first stage of processing in order to obtain a signal that clearly identifies the peak that characterizes the systolic contraction, the phase of the cardiac cycle in which blood is being forced into the arteries. This procedure was performed according to the method described in [36], which involves decomposing the signal into different levels of detail, followed by a denoised reconstruction of the original signal. This type of processing allows for the characterization of a PPG signal, especially in those cases where the level of luminosity around the sensor and possible involuntary movements of the user have generated noise during the data acquisition, considering that the measurement points defined on the fingers and toes can be prone to this type of noise sources.

**Figure 5:**
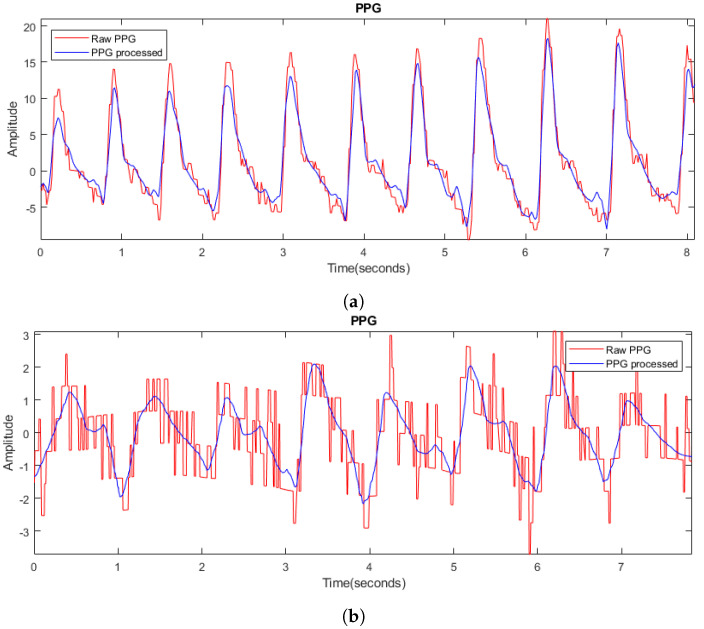
(**a**) Original signal of PPG 1 (in red) and its result after processing (in blue); (**b**) original signal of PPG 2 (in red) and its result after processing (in blue).

Figure 5a shows the initial result of the PPG signal processing, highlighted in red, together with the reconstructed signal, highlighted in blue. An improvement in signal clarity is observed, which facilitates identification of the systolic contraction point. On the other hand, Figure 5b shows an initial signal with significant noise, highlighted in red, but the reconstruction result clearly describes the original signal, being clear and of sufficient quality for further analysis. These two extreme cases illustrate the ability of the processing to improve the quality of the signal from a clean initial signal to one with a high level of noise. This processing is different from traditional smoothing techniques, which are based on a smoothing factor that can vary from case to case, making it difficult to adapt the algorithm to different situations and not guaranteeing effective signal denoising.

### 2.4. HRV and PTT Calculation

After processing the ECG and PPG signals, the characteristic points of these signals are extracted to determine the HRV and PTT parameters. For this purpose, the R-peaks in the ECG signal and the systolic amplitude in the PPG, called the S-point in this context, are identified.

An algorithm has been developed to identify the peaks of each signal. In the case of the ECG, the algorithm begins to evaluate the signal and detects an R-peak when a predefined threshold based on the signal magnitude is exceeded. In addition, a minimum time interval between consecutive peaks is set to ensure accurate identification. This information is used to calculate HRV, expressed as the approximate number of beats per minute. For the PPG signal, the algorithm uses the frequency of the R-peaks identified in the ECG to estimate the time of occurrence of each S-peak. This information is correlated because they represent the same phase of the cardiac cycle.

**Figure 6:**
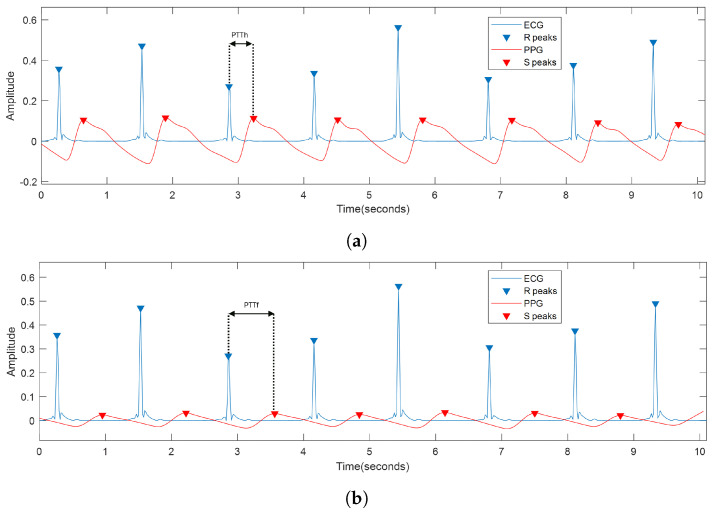
(**a**) ECG and PPG signal measured on the hand. Identification of the R- and S-peaks; (**b**) ECG and PPG signal measured on the foot. Identification of R- and S-peaks.

Because the ECG and PPG signals are acquired simultaneously, the PTT parameter can be determined as the time difference between the S-point on the PPG and the preceding R-peak on the ECG. Therefore, $PTT_{h}$ and $PTT_{f}$ are defined as the pulse transit time from when the blood leaves the heart to when it is reflected in the fingers and toes of the hand, respectively. Figure 6 shows the result of processing an ECG signal in blue and PPG in red, together with an example of the result of identifying R- and S-points, the latter measured on both the hand and foot. In addition, the graphs illustrate the $PTT_{h}$ and $PTT_{f}$ parameters.

### 2.5. Pulse Wave Velocity

Once the time it takes for the blood pulse wave to travel from the heart to the fingers and toes has been determined, it is essential to calculate the distance traveled to determine the pulse wave velocity.

#### 2.5.1. Arterial Length

Calculating the exact length of the arteries and capillaries that make up the circulatory system is not an easy task. Although it is possible to physiologically describe the arterial tree and make an approximate estimate of its length, this measurement would not be completely accurate due to anatomical variations between individuals. Previous studies, such as [18], establish a mathematical model between people’s height and arterial lengths. The formulas developed take into account the length of the brachial artery to the elbow and the distance from the heart to the ankle. However, these formulas are not applicable in this work because the measuring points do not coincide. Therefore, it is proposed to find a new mathematical relationship to estimate the approximate length of the arteries and blood vessels that carry blood from the heart to the fingers and toes.

The route of blood from the heart to the fingers is through the axillary artery, which then continues to the brachial artery and reaches the ulnar and radial arteries, where it is finally distributed to the blood vessels and capillaries of the fingers. The path to the toes, on the other hand, is more extensive, starting from the aorta, which bifurcates into the right and left iliac arteries of each lower extremity and then passes through the tibial arteries until it reaches the dorsalis pedis and plantar arch, which are responsible for carrying the blood flow to the vessels of the toes. Once the blood path has been identified, this study proposes three lengths to estimate these distances according to each side of the body. The lengths of the left and right arteries and the plantar artery represent the distance from the heart to the hallux of each foot.

- Right/Left arm artery: The length of the brachial arteries is measured from the end of the left clavicle attached to the sternum to the tip of each index finger.
- Right/Left artery: The set of arteries that carry blood from the heart to the heel. The distance is measured from the end of the left clavicle, similar to the previous case, directly to the heel.
- Plantar artery: The length is measured from the heel to the midpoint of the hallux.

**Figure 7:**
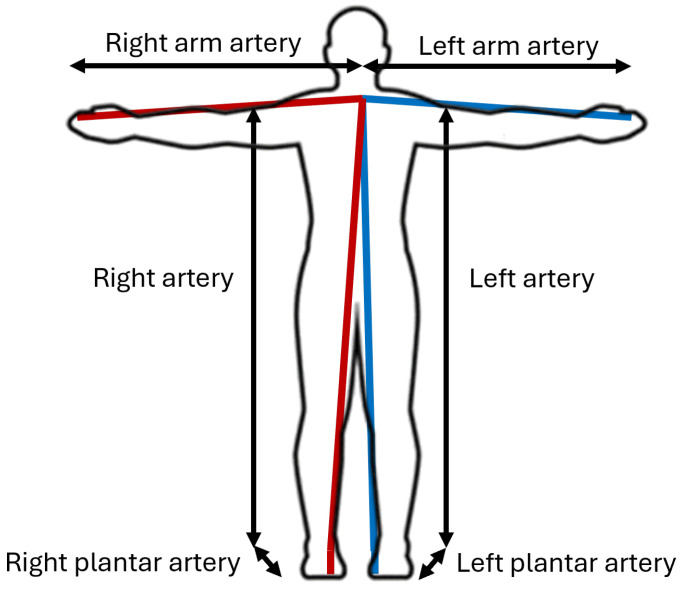
Proposed distances for the arteries on each side of the body. The right side is shown in red, and the left side is shown in blue.

Figure 7 illustrates a schematic of the proposed measurements to characterize the distances traveled on each side of the body, with the right side highlighted in red and the left side highlighted in blue.

Arterial length data were collected from a group of 35 individuals of different ages, sexes, heights, and physical characteristics. A least-squares fit was performed on these data to achieve a mathematical optimization that allowed for an automatic approximation of arterial lengths as a function of height in meters (m). This process resulted in the estimation of the coefficients of a continuous function that models and approximates the data. Five different equations were established to describe the lengths of the right (RA) and left (LA) arm arteries, the right (AR) and left (AL) arteries, and the plantar artery (PA), all as a function of height (*H*).

(1) $$RA=-0.5263H^{2}+2.201H-1.311$$

(2) $$LA=-0.5018H^{2}+2.083H-1.245$$

(3) $$AR=-0.3993H^{2}+1.967H-0.8949$$

(4) $$AL=-0.5543H^{2}+2.488H-1.336$$

(5) $$PA=-0.2921H^{2}+1.055H-0.736$$

To calculate the total distance from the heart to the hallux of each foot, it is necessary to sum the lengths of the right (AR) and left (AL) arteries together with the measurement of the plantar artery (PA). Consequently, the parameters $AR_{f}$ and $AL_{f}$ are defined to characterize these combined distances.

#### 2.5.2. PWV Calculation

Once the distances and PTT to the measurement points of the PPG signals have been determined, it is possible to define PWV as the quotient between these two parameters, expressed in units of meters per second (m/s). Therefore, the PWV to the toe ($PWV_{f}$) and the PWV to the fingertip ($PWV_{h}$), both on the right and left side, are determined according to the following expressions:

(6) $$PWV_{rf}=\frac{AR_{f}}{PTT_{rf}}$$

(7) $$PWV_{lf}=\frac{AL_{f}}{PTT_{lf}}$$

(8) $$PWV_{rh}=\frac{RA}{PTT_{rh}}$$

(9) $$PWV_{lh}=\frac{LA}{PTT_{lh}}$$

Where $PTT_{rf}$ and $PTT_{lf}$ are the time between the R-peak of the ECG and the S-peak of the PPG measured at the right and left toes, respectively. $PTT_{rh}$ and $PTT_{lh}$ are the time to reach the right and left fingertips, respectively.

### 2.6. ABI Calculation

In this study, it is proposed to relate the PWV to determine the ABI. For this purpose, the ABI is defined as the quotient between the PWV measured up to the foot and the PWV obtained up to the hand on both sides of the body, expressed by the following equations.

- Right ABI:
  (10) $$ABI_{r}=\frac{PWV_{rf}}{PWV_{rh}}$$
- Left ABI:
  (11) $$ABI_{l}=\frac{PWV_{lf}}{PWV_{lh}}$$

The proposed ABI calculation is performed for each measurement point, i.e., for each ECG R-peak in relation to the next PPG S-peak, both in the hand and in the foot. In this way, a specific number of measurements is obtained according to the amount of data available.

The measurement system is complemented by a desktop software application for computers developed in the Matlab App Designer tool [37].This programming includes the creation of a graphical user interface (GUI), as well as the necessary algorithms for signal processing, including the calculation of the ABI according to the methodology proposed in this work. The final ABI value corresponds to the arithmetic mean of the data evaluated at 30-s intervals.

The GUI provides a visualization of the acquired ECG and PPG signals in addition to a slider that allows for the selection of the time period to analyze the data. This is useful in case involuntary movements have affected the measured signals.

## 3. Results

### 3.1. System Validation

The validation of the system included the assessment of its accuracy compared with the conventional ABI measurement method. The study included 22 subjects: 13 men and 9 women, aged between 46 and 86 years (mean age 64.3 years), diagnosed with diabetes mellitus and at risk for PAD. The study was conducted at the San Juan Medical Center in Alicante, Spain, with the approval of the Ethics Committee of the Health Department of the General Hospital of Alicante. Before the start of the examination, each participant signed an informed consent form to participate in the study. During the medical evaluation, general information was collected, including age, body weight, smoking habits, and diet.

The examination of each patient began with a vascular examination to measure the ABI using the traditional method. Subsequently, the evaluation was performed with the system developed in this work. The experimental protocol included a rest period of 5 to 10 mins before each test, with the patient being in the supine decubitus position.

#### 3.1.1. ABI Measurement by Vascular Exploration

**Figure 8:**
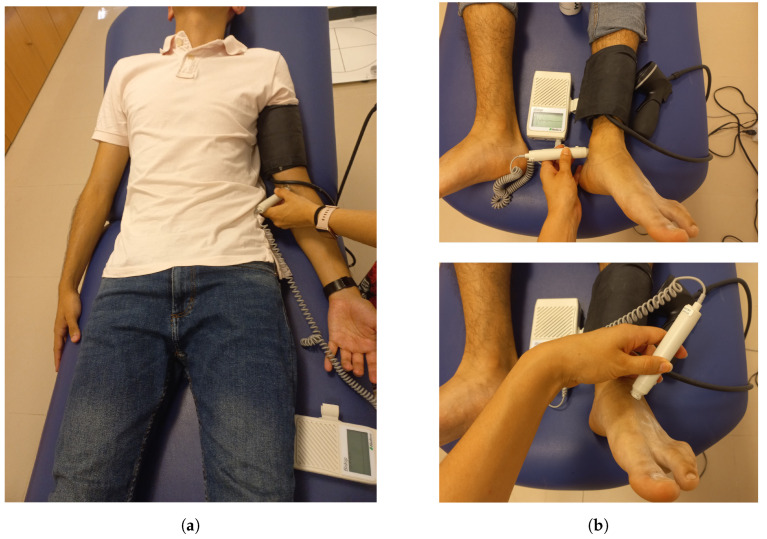
(**a**) Location of the brachial artery in the arm; (**b**) locations of the dorsalis pedis and posterior tibial arteries.

The ABI measurement procedure by vascular exploration was performed by medical personnel specialized in podiatry. It was performed using a sphygmomanometer with pressure cuff and a Bidop V3 vascular Doppler device. The patient was placed in the supine decubitus position, and the pressure cuff was placed above the elbow flexion point to locate the brachial artery. The Doppler equipment was then turned on, and SBP was recorded based on the sounds generated across the artery due to pressure changes. The same procedure was repeated on both ankles, with the cuffed sphygmomanometer placed over the malleoli and SBP measured over the pedal artery at the instep and posterior tibial artery behind the ankle. Figure 8 shows an example of the device setup and arterial locations.

#### 3.1.2. ABI Measurement Using the Developed System

The ECG and PPG signals are recorded simultaneously, starting with one pair of limbs, either the right or left hands and feet, and then moving to the other set. Similar to the conventional method, the patient must remain lying down. The patient’s height is recorded, and the two electrodes necessary for the acquisition of the ECG signal are placed, one in the left axillary region and the other on the line dividing the pectoral muscles. Next, the cables that facilitate the transmission of the signal from the electrodes to the acquisition device are connected, following the arrangement of the ECG_P and ECG_N inputs. In order to obtain the PPG signals, it is essential to ensure the correct positioning of the fingers so that the sensor is completely covered and there is optimal contact between the fingertip and the device, without exerting excessive pressure. This is performed according to the recommendations in Section 2.2.3. Signal acquisition is performed for 90 s on each side of the body.

**Figure 9:**
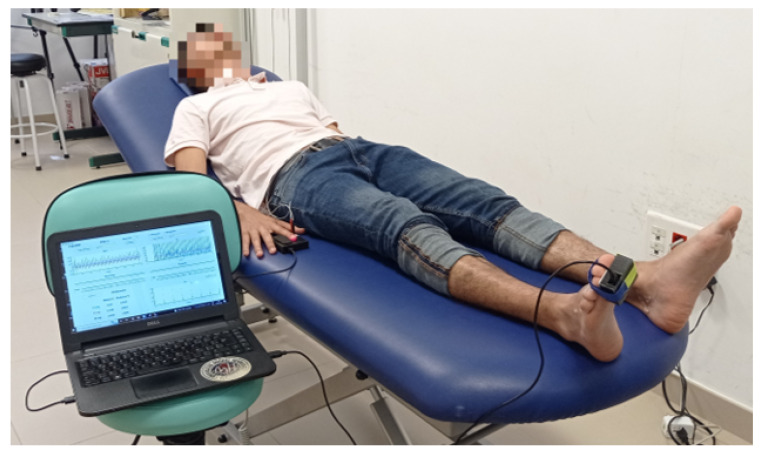
Subject in supine position and configuration of the measurement acquisition system and a computer with the data display interface.

Once acquired, the data are visualized and processed in the developed application hosted on a computer. This application displays the corresponding ABI value. It is important to note that this result is the arithmetic mean of the data collected over a period of at least 30 s. Figure 9 shows an individual in the supine position and the setup configuration for acquiring measurements with the developed system and a computer where the data are stored and which has the GUI for its visualization.

### 3.2. Validation Results

During the medical evaluation, additional information was collected for each patient, including clinical variables such as body weight, time since diagnosis of diabetes, and smoking habits, which may be related to the degree of PAD.

To facilitate the analysis of the results, Table 1 presents the data collected from the 22 subjects who participated in the validation study. The information is presented by sex and includes the ABI obtained with the traditional method and with the system developed in this work, together with data related to age, body weight and height.

**Figure 10:**
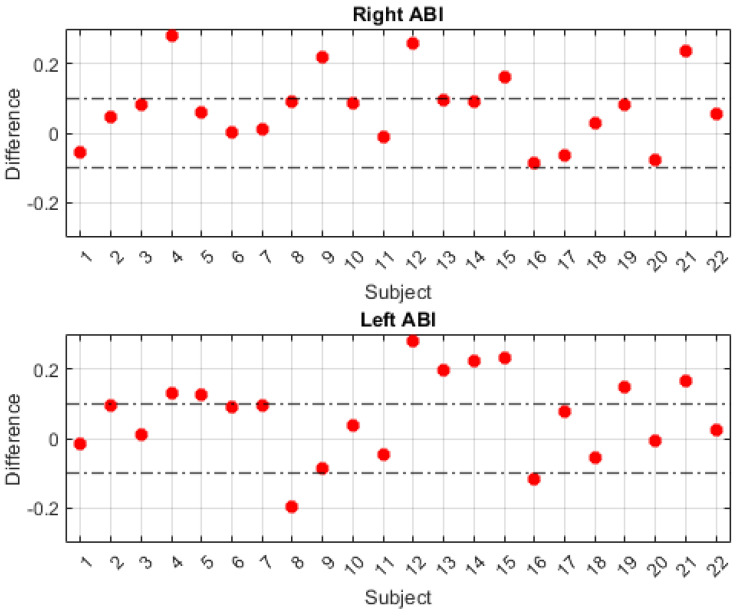
Comparison of right and left ABI measurements obtained with the traditional method and with the system developed in this work.

In order to improve the visualization of the obtained ABI results, it is suggested to generate a comparative graph of the results obtained with the two calculation methods. This comparison is shown in Figure 10. The abscissa axis represents the 22 patients, and the ordinate axis represents the difference between the ABI results obtained with the traditional method and those obtained with the developed system. In order to evaluate the degree of agreement between the two measurement methods, a range of ±0.1 difference is established to determine the degree of accuracy and variability of the proposed system. In the graphs, a horizontal line is included to delimit the defined difference range, which facilitates the identification of the number of results within and outside the established tolerance interval. The upper part of the figure represents the difference between the data obtained from the right ABI, while the lower part shows the difference between the data obtained from the left ABI.

According to the validation results presented in Table 1 and Figure 10, a total of 17 right ABI measurements obtained from the subjects were found to be within the established tolerance interval, representing 77.3% of the total. With respect to the left ABI results, 12 measurements were found to be within the concordance interval of ±0.1, representing 54.54% of the total subjects. Overall, when considering measurements from both sides of the body, a total of 29 measurements, representing 65.9% of subjects, were found to be within the established precision interval.

In terms of sex, the results show that for female subjects, 77.78% of the ABI measurements on the right side had a difference of less than 0.1, while this percentage was 66.67% on the left side. For male subjects, 76.92% of the ABI measurements on the right side had a difference of less than 0.1 with respect to the standard, while this value was 46.15% on the left side.

**Figure 11:**
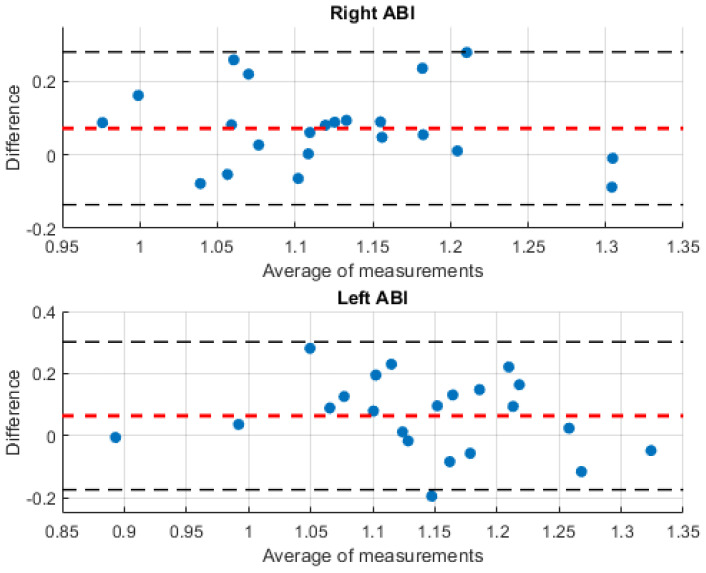
Bland–Altman plot comparing the ABI obtained with the traditional method and the ABI calculated with the developed system. The red lines represent the mean of the differences between the methods, while the black lines indicate the limits of agreement, the blue dots represent the difference between the results obtained with the two methods compared to the mean of these results.

On the other hand, the Bland–Altman method [38] is a graphical tool that is widely used to compare two measurement methods, allowing their accuracy to be evaluated and providing a relevant clinical interpretation. This type of graph shows the difference between the results obtained with the two methods compared with the mean of these results [39]. The horizontal lines of the graph represent the mean of the differences and the limits of agreement, which are defined as 1.96 times the standard deviation of the mean difference. This range of agreement provides a frame of reference within which most of the differences between measurements made with the two methods are expected to be found. In this context, the application of this method is essential for evaluating the reliability and appropriateness of the results obtained in this study, especially with regard to the potential clinical application of the developed system. Figure 11 illustrates the Bland–Altman graphical analysis for this study based on measurements performed on both sides of the body. In this graph, the red lines represent the mean of the differences between the methods, while the black lines indicate the limits of agreement.

## 4. Discussion and Conclusions

This study presents a system consisting of two electronic devices that allow for the simultaneous acquisition of ECG and PPG signals to calculate PWV and thus determine the ABI value on each side of the body. The PPG signals are recorded from both hands and feet. These devices are designed to be portable and non-invasive to ensure patient comfort and ease of use in both clinical and home settings. The MAX86150 sensor was selected for the design because of its high performance and accuracy in obtaining ECG and PPG measurements. This sensor offers an advanced manufacturing design that ensures efficient acquisition of the signal spectrum, which simplifies post-processing. For the signals, an algorithm has been developed to identify the characteristic parameters related to the ECG R-peak and the systolic amplitude of the PPG signal. This algorithm is divided into two distinct stages: in the first stage, a high-pass filter is applied to remove any measurement noise induced by the electrical signal; then, in the second stage, the wavelet transform is used for signal reconstruction and noise removal. There are several signal processing techniques that have proven effective in pattern recognition and arrhythmia identification in electrocardiogram (ECG) signals. Among these techniques are the application of wavelet transforms and machine learning methods [40,41]. In the context of this work, these techniques could be used. However, the signal decomposition strategy and the application of the wavelet transform ’sym4’ proved to be simple and robust enough for the analysis of ECG signals. This second step was fundamental to validate the processing of different lead signals obtained in the ECG as well as to evaluate the cases in which the pulses are weak. Although the proposed system is designed for ambulatory and clinical use and with patients at rest, it could be affected by possible interferences or movements of the patient. Therefore, a signal processing approach divided into two sections has been chosen to mitigate such problems.

This study proposes a set of three distances that describe the trajectory of blood through the arteries to the fingers and toes. The goal is to achieve a mathematical optimization that automatically estimates the arterial lengths as a function of each individual’s height. The time and distance from the heart to the PPG measurement points are calculated to obtain the corresponding PWV. In addition, the ABI is defined as the ratio of the PWV obtained at the foot to the PWV obtained at the hand.

After highlighting some of the design features of the developed system, the results of the experiments performed to validate the system at the medical level are discussed. For this purpose, the results obtained are compared with those of the traditional clinical procedure, which has been established as the gold standard for the diagnosis of PAD [6]. The results of the 22 patients who participated in the experiment are detailed in Table 1. In addition, the graphs in Figure 10 are used to compare the concordance results obtained for each patient. A variation interval between −0.1 and +0.1 was defined to analyze the degree of agreement of each result. This interval is defined to evaluate the accuracy of the developed system in comparison with the gold standard used. Because the intervals defining the diagnosis are very sensitive values that can lead to misdiagnosis, it has been considered that the results obtained with the proposed system, which present values lower than 0.9 and higher than 1.3 with a variation of ±0.1 with respect to the traditional procedure, are not considered dangerous, at least in this first medical validation study.

Figure 10 shows two graphs that illustrate the correlation of the errors between the measurements obtained with the standard method and those obtained with the system proposed in this work. The graphs differentiate the number of results that fall within the acceptable evaluation range established in this research. Of the 44 results, 29 (66%) were within this range. However, upon closer analysis of the results obtained with the proposed system, it was observed that the mean absolute error (MAE) of the 44 measurements, considering both sides of the body, was 0.1051. This value indicates a fit very close to 0.1, which supports the results of previous studies that have already demonstrated the feasibility of implementing PPG signals for ABI measurement at the clinical level [16]. Considering the sex of the participants, 72.22% of the results obtained from female participants fell within the agreement interval of ±0.1. The MAE for the differences between the measurements was 0.0939, which is less than 0.1, indicating significant agreement between the measurements using both methods. This initial study suggests that the system developed in this work may help to improve the diagnosis of PAD in women. This is particularly relevant given the observed increase in cases in recent years [42]. For the male participants, the results show that 61.54% of the measurements fell within the range of agreement, with an inter-measurement MAE of 0.1129. Although the mean variation of the measurements is slightly above 0.1, increasing the sample size and improving the adaptation of the arterial lengths would increase the sensitivity and accuracy of the developed system.

The results derived from the Bland–Altman graphical analysis, Figure 11, show that for both the right and left ABI, the estimation errors of the results obtained with the proposed system with respect to the standard measurements were distributed within the 95% confidence interval. In both cases, 21 out of 22 measurements were within this interval, indicating satisfactory agreement. Furthermore, the Root Mean Square Error (RMSE) is 0.3396 for the right ABI agreement analysis and 0.2991 for the left ABI. This statistical analysis highlights the consistency and accuracy of the proposed system in assessing ABI compared to the standard method, which reinforces its clinical validity and potential usefulness in healthcare settings. Regarding the atypical result observed in a subject outside the confidence interval, it is important to note that his clinical history reveals complications in his cardiac system that affect blood circulation through the arteries. Therefore, it is important to perform an evaluation of a group of subjects with similar cardiac problems to investigate and better understand how these conditions may affect the reliability of the results. This further investigation will identify strategies to improve the accuracy and interpretation of results in patients with cardiovascular complications, which will strengthen the validity and usefulness of this study in a clinical context.

In the case of subject 11, it was not possible to obtain a quantitative value of the patient’s arterial health during vascular exploration using the standard method. This finding was associated with a high degree of arterial obstruction, suggesting the presence of arterial calcification, a common condition in diabetic patients with chronic kidney damage [43]. In this context, in these types of patients, the ABI value is expected to be higher than 1.3, indicating arterial incompressibility in the lower extremities [44]. However, the system developed in this work allowed for the determination of a quantitative value of the subject’s arterial health status. The results showed a value greater than 1.3 on both sides of the body, as shown in the results table. The ABI value reported by the standard method was assumed to be >1.3. This finding suggests that the system may be useful in evaluating patients with arterial obstruction and calcification.

It is worth noting that there are currently no medical validation studies in the scientific literature that establish the relationship between PWV and ABI as investigated in this study. The results demonstrate a high degree of agreement and accuracy, suggesting that PWV may be clinically useful in improving the diagnostic process for PAD, particularly in primary care settings. This finding is a notable improvement as it has the potential to alleviate the difficulties associated with conventional vascular assessment methods. The measurement of PWV, a non-invasive and simple technique, could reduce the time needed to diagnose PAD, benefiting patients and healthcare providers alike.

Future research should aim to validate these findings in a larger and more diverse population. In addition, the feasibility of integrating PWV measurement to determine ABI into routine clinical practice should be evaluated.

On the other hand, the integration of artificial intelligence techniques to adapt the model to the individual characteristics of each subject may be beneficial to improve the accuracy in estimating the distances traveled by blood flow in the arteries. This strategy could reduce discrepancies between model predictions and clinical data.

## Figures and Tables

**Table 1:** Subject information and ABI measurement results using the traditional method and developed system.

Subject	Sex	Age	Body Weight (Kg)	Height (m)	Traditional Method		Developed System	
					Right ABI	Left ABI	Right ABI	Left ABI
1	Female	77	76	1.535	1.03	1.12	1.083	1.137
2		61	59	1.53	1.18	1.20	1.132	1.104
3		46	93	1.64	1.10	1.13	1.018	1.118
4		56	93.5	1.63	1.35	1.23	1.071	1.099
5		52	46.5	1.465	1.14	1.14	1.079	1.014
6		66	75.3	1.48	1.11	1.11	1.107	1.021
7		74	60.5	1.55	1.21	1.26	1.199	1.166
8		79	75.2	1.49	1.17	1.05	1.081	1.245
9		64	70	1.535	1.18	1.12	0.960	1.204
10	Male	54	87	1.64	1.02	1.01	0.932	0.974
11		73	71	1.63	>1.30	>1.30	1.309	1.348
12		47	83.1	1.875	1.19	1.19	0.931	0.909
13		49	79	1.79	1.18	1.20	1.066	1.005
14		86	72	1.65	1.20	1.32	1.110	1.099
15		75	96.5	1.64	1.08	1.23	0.918	1.000
16		67	57.8	1.78	1.26	1.21	1.348	1.326
17		64	108.7	1.71	1.07	1.14	1.134	1.061
18		48	66	1.73	1.09	1.15	1.063	1.207
19		55	79.3	1.68	1.16	1.26	1.079	1.112
20		66	91	1.695	1.00	0.89	1.078	0.896
21		83	93.5	1.61	1.30	1.30	1.064	1.136
22		73	53.5	1.66	1.21	1.27	1.155	1.246

## Data Availability

Data are contained within the article.
